# Biomarker MicroRNAs for Diagnosis of Oral Squamous Cell Carcinoma Identified Based on Gene Expression Data and MicroRNA-mRNA Network Analysis

**DOI:** 10.1155/2017/9803018

**Published:** 2017-09-17

**Authors:** Hui Zhang, Tangxin Li, Linqing Zheng, Xiangya Huang

**Affiliations:** ^1^Guanghua School of Stomatology, Affiliated Stomatological Hospital, Guangdong Province Key Laboratory of Stomatology, Guangdong, China; ^2^Oral and Maxillofacial Center, Kiang Wu Hospital, Macau

## Abstract

Oral squamous cell carcinoma is one of the most malignant tumors with high mortality rate worldwide. Biomarker discovery is critical for early diagnosis and precision treatment of this disease. MicroRNAs are small noncoding RNA molecules which often regulate essential biological processes and are good candidates for biomarkers. By integrative analysis of both the cancer-associated gene expression data and microRNA-mRNA network, miR-148b-3p, miR-629-3p, miR-27a-3p, and miR-142-3p were screened as novel diagnostic biomarkers for oral squamous cell carcinoma based on their unique regulatory abilities in the network structure of the conditional microRNA-mRNA network and their important functions. These findings were confirmed by literature verification and functional enrichment analysis. Future experimental validation is expected for the further investigation of their molecular mechanisms.

## 1. Introduction

Oral squamous cell carcinoma (OSCC) is the sixth most common cancer with more than 300,000 cases worldwide each year [1]. It is the most malignant tumor in the oral and maxillofacial regions and accounts for 90% of oral cancers [2, 3]. The risk factors for OSCC could be tobacco, alcohol consumption, betel quid (BQ) chewing, Bidi smoking, and genetic predisposition [4, 5]. OSCC can metastasize to lymph-node, even to remote organs with high mortality rate. The present diagnosis of OSCC often happened at late stage and the treatment can be unsuccessful due to its local recurrence. The precise early diagnosis is critical and essential to the future prevention and personalized treatment of this disease.

MicroRNA is a family of functional noncoding RNA molecules containing about 22 nucleotides, which play roles in the posttranscriptional gene regulation. Since many key biological processes including the development, differentiation, and cell cycles are regulated by microRNAs, the abnormal expression of microRNAs is often associated with the initialization and progression of many diseases [6, 7]. Thus miRNAs usually could serve as suitable biomarkers for many diseases, such as neurodevelopmental disorders [8], cancer, and cardiovascular disease [9–11].

Previous studies have demonstrated that microRNAs played important roles in OSCC. For example, microRNA-23b/27b cluster is reported as tumor suppressive and regulates the MET oncogene in OSCC [12]. MicroRNA-27a-3p can regulate transition from epithelial to mesenchymal in OSCC by targeting YAP1 [13]. The apoptosis-related protein expression and radiosensitivity in BQ-associated OSCC are regulated by microRNA-17-5p [14]. Metabolic shift in OSCC is mediated by microRNA-340 targeting glucose transporter-1 [15]. Tumor growth and activation of NF-*κ*B signaling were promoted via the regulation of NLK by microRNA-92b in OSCC [16]. In addition, microRNA-17/20a was suggested as a prognostic marker since it can inhibit cell migration in OSCC [2]. Circulating microRNA-21 and PTEN (phosphatase and tensin homolog) are reported as promising biomarkers for detection of OSCC [17].

From the above introduction, we believe that microRNAs are good candidates to act as diagnostic and prognostic biomarkers of OSCC. As we know, OSCC is a complex and heterogeneous disease. For this reason, more precise and personalized biomarkers are needed for the diagnosis, prognosis, and treatment of OSCC. Until now, very few studies have focused on the expression data of OSCC to integrate it with the microRNA-mRNA network structural analysis for biomarker discovery in OSCC, especially the application of bioinformatics and network analysis to the study of the functions of microRNAs in the OSCC initialization and progression.

The experimental methods for biomarker discovery are time-consuming and costly. Bioinformatics screening will be helpful to the efficient biomarker screening. Previously, several models have been developed to infer key and biomarker microRNAs in complex diseases from conditional gene expression data. Differential expression genes (DEGs) are often used to screen biomarker genes, but only few DEGs are validated as biomarkers; therefore integrative analysis of DEGs with other information is very necessary for efficient biomarker discovery. As described in previous work [18–21], these models screen the potential biomarker based on the scrutinizing of the structure of the conditional microRNA-mRNA network. By statistical analysis of the network structure and functions of the biomarker microRNA's targets, the model can very effectively identify novel putative microRNA biomarkers for the diagnosis of complex diseases. So we here apply the model to the biomarker microRNA discovery for diagnosis of OSCC.

## 2. Materials and Methods

**Figure 1:**
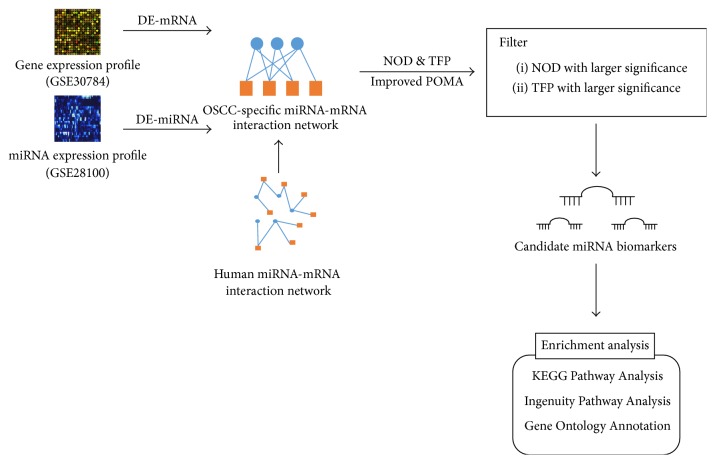
Schematic pipeline for the identification of oral squamous cell carcinoma (OSCC) microRNA biomarkers.

The schematic pipeline of the present work for the data collection, model construction, biomarker microRNAs prediction, and validation and enrichment analysis of the targets of the predicted microRNAs is presented in Figure 1. The details of the step-by-step procedures for the screening of OSCC diagnostic biomarker microRNAs are described as follows.

### 2.1. Gene Expression Data Collection

The data for the OSCC gene expression and microRNA expression were extracted from the GEO database [22]. The OSCC associated expression data in the GEO database are listed in Table 1. Eight OSCC associated data sets measured from different microarray platforms are available in the GEO database. After the condition filtering, the final data used for the construction of OSCC-specific microRNA-mRNA network are GSE30784 and GSE28100. The former is the mRNA expression data including 167 OSCC samples and 45 samples as control [23] and the latter is the microRNA expression data with 17 OSCC samples and 3 control samples [24]. The data were normalized and the differentially expressed mRNAs were identified based on linear models in Limma R package [25, 26]. The *p* value and other parameters were calculated with the empirical Bayes (eBayes) method. The Benjamini-Hochberg correction was applied to adjust the *p* values. The adjusted *p* value less than 0.05 was chosen as the cut-off criteria.

The reported OSCC associated microRNAs were also collected from PubMed citations by the search criteria “(Oral squamous cell carcinoma OR OSCC) AND (miRNA OR microRNA) AND (biomarker^*∗*^ OR marker^*∗*^)”. They were checked manually and listed in Table 2.

### 2.2. Prediction of microRNA Biomarkers for Diagnosis of OSCC

As reported in the previous researches in Shen's group [18–21], two measurements are important for candidate biomarker microRNA. The first one is the novel of degree measurement (NOD). It measures the number of genes solely targeted by certain microRNA [19, 20]. This character is reasonable since the abnormal alteration of this type of interaction cannot be compensated by another microRNA-mRNA interaction pair as most of the microRNA-mRNA interactions are synergic. The other measurement is the transcription factor percentage (TFP), which was defined as the percentage of transcription factor (TF) genes of all targets of the microRNA [18].

According to the above hypothesis, the OSCC-specific microRNA-mRNA network was constructed by mapping the detected differentially expressed microRNAs in OSCC onto the reference human microRNA-mRNA network. The reference network was constructed with the integration of the experimentally validated and computational predicted microRNA-mRNA pairs. The experimentally validated data included information from miRecords [27], TarBase [28], miR2Disease [29], and miRTarBase [30], while the computational predicted microRNA-mRNA pairs are extracted from no fewer than 2 databases among HOCTAR [31], ExprTargetDB [32], and starBase [33]. With the reconstructed conditional network, the above-mentioned measurements, that is, the NOD and TFP, were calculated for each microRNA in the OSCC-specific network. MicroRNAs with significantly large NOD and TFP values (Wilcoxon signed-rank test, *p* value < 0.05) were screened as putative biomarkers.

### 2.3. Functional Enrichment Investigation of the Targets of Predicted OSCC Diagnostic MicroRNA Biomarkers

Functional enrichment analysis of the genes targeted by the identified candidate biomarker microRNAs from the OSCC-specific microRNA-mRNA networks was performed through three different tools: Gene Ontology Annotation, KEGG Pathway Analysis, and Ingenuity Pathway Analysis (IPA). Here, the first two analyses were conducted on the DAVID (Database for Annotation, Visualization, and Integrated Discovery) online analysis webpage [34]. The significantly enriched pathways and ontologies for OSCC with *p* value less than 0.05 were ranked. The *p* value was calculated based on the hypergeometric test and FDR adjustment was used for multiple test correction.

## 3. Result and Discussion

### 3.1. The Characterization of the Previous Reported OSCC Diagnostic Biomarker MicroRNAs

We checked the PubMed citations and the previously reported biomarker microRNAs for OSCC were listed in Table 2. From Table 2, it is clear that all the reported microRNAs have high NOD and TFP values except miR-21-3p, which cannot be extracted from the reconstructed microRNA-mRNA network. This observation confirmed that the model using the NOD and TFP as two measurements for the evaluation of the potential biomarkers is applicable for OSCC biomarker discovery.

### 3.2. Predicted Diagnostic Biomarker MicroRNAs for OSCC

We first identified 56 dysregulated microRNAs and 3375 differentially expressed genes in OSCC by using the pipeline presented in Figure 1. Five microRNAs were identified through Wilcoxon signed-rank test with *p* value less than 0.05. These microRNAs were predicted to be candidate biomarkers for the diagnosis of OSCC. Their network structural characteristics in the microRNA-mRNA network, including the number of targets and NOD and TFP values calculated based on the conditional OSCC-specific microRNA-mRNA network, are listed in Table 3. Among the five microRNAs, miR-155-5p was reported in previous work as biomarker [35, 36]. The other four microRNAs, that is, miR-148b-3p, miR-629-3p, miR-27a-3p, and miR-142-3p, are the novel putative biomarkers identified for OSCC.

### 3.3. Literature-Based Validation of Identified MicroRNA Biomarkers

**Figure 2:**
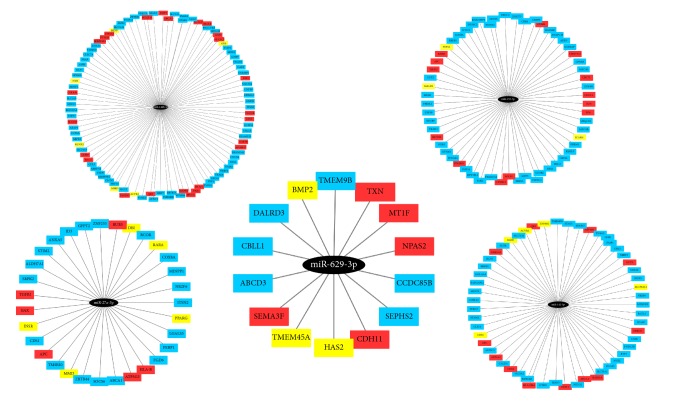
Candidate oral squamous cell carcinoma (OSCC) microRNA biomarkers with their target genes. Here, ellipses in black represent 5 microRNA biomarkers. Genes in red are associated with OSCC according to literature reports while genes in yellow are reported to be associated with other oral diseases.

The targets of the five putative microRNAs are presented in Figure 2. From the figure, we can see that some of the targets of these microRNAs have been reported to be associated with OSCC (genes colored red) or other oral diseases (genes colored yellow) according to the PubMed citations.

### 3.4. Functional Enrichment Analysis of Target Genes of Candidate MicroRNA Biomarkers

**Figure 3:**
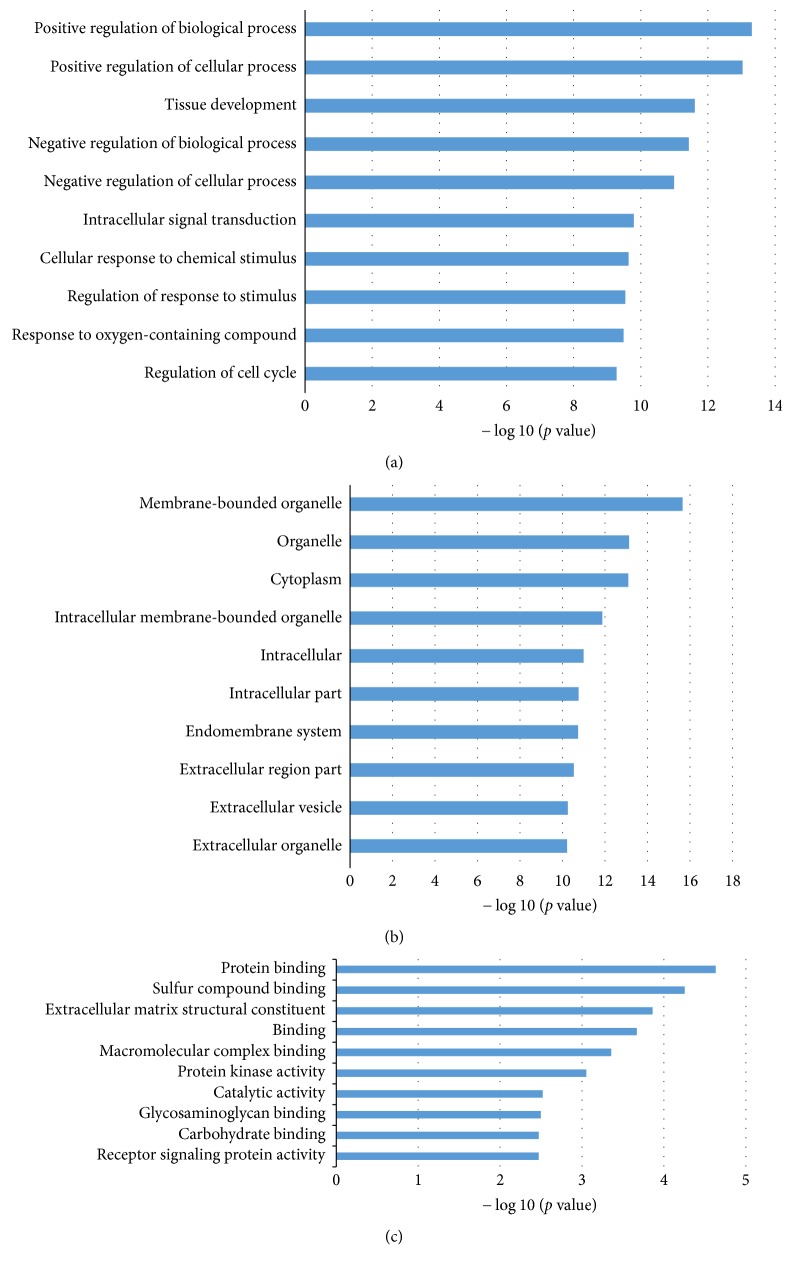
Gene Ontology (GO) Annotation for genes targeted by identified microRNA biomarkers. (a), (b), and (c), respectively, represent three levels of GO: biological process, cellular component, and molecular function. The statistical significance value (*p* value) has been negative 10-based log-transformed. The top 10 significantly enriched items are listed for each level.

The functional enrichment analysis was further performed to explore the roles of the uniquely regulated genes of the identified microRNAs in OSCC with DAVID and IPA tools.  Figure 3 presented the Gene Ontology (GO) Annotation for targets of the identified microRNA biomarkers in OSCC. The three levels of GO analysis are presented in Figures 3(a)–3(c), respectively, for biological process, cellular component, and molecular function. The top 10 significantly enriched items are listed for each level. Most of the dysregulated biological processes are the positive/negative biological or cellular processes, the regulation of cell cycle, and the response to oxygen-containing compound. The former are well-known popular cancer-associated processes, while the latter is associated specifically with OSCC [37–39]. The most enriched molecular functions are the general cancer-associated items, such as protein binding, protein kinase activity, and receptor signaling protein activity. The functions for carbohydrate binding [40–42] and glycosaminoglycan binding [43, 44] were also discovered in the OSCC studies.

**Figure 4:**
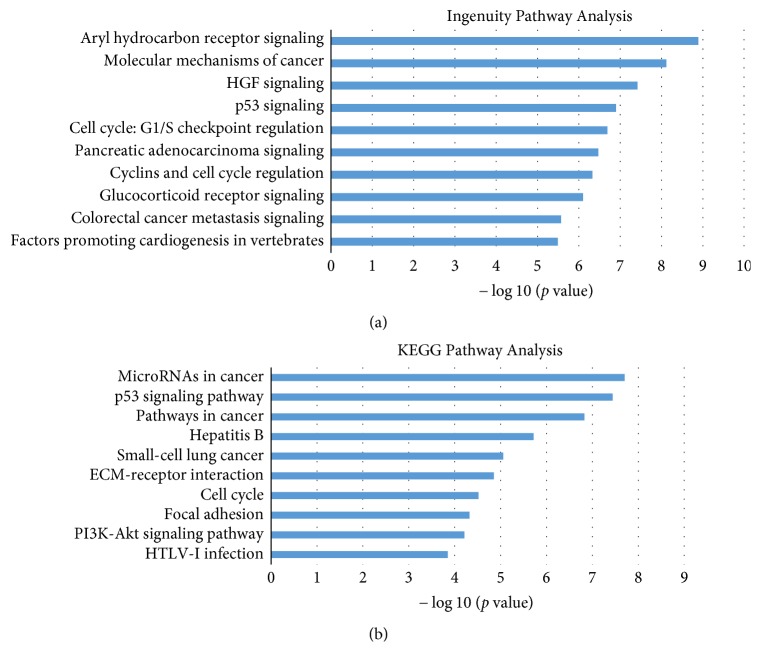
KEGG Pathway Enrichment Analysis and Ingenuity Pathway Analysis for genes targeted by identified microRNA biomarkers. The statistical significance value (*p* value) has been negative 10-based log-transformed. The top 10 significantly enriched pathways are listed, respectively, in (a) and (b).

The result of the pathway enrichment analysis of the targeted genes of the putative microRNA biomarkers is displayed in Figure 4. The most common cancer-associated pathways like p53 signaling pathway and cell cycle pathway are enriched in both the DAVID and IPA methods. There are still other pathways such as PI3K-Akt signaling pathways and colorectal cancer metastasis were screened by these two enrichment analyses. The Aryl hydrocarbon receptor [45–47], the HGF [48, 49], ECM receptor interaction [50, 51], Hepatitis B [52, 53], and glucocorticoid receptor signaling [54] are all supported by the PubMed citations.

## 4. Conclusions

In this research, we applied the concepts of NOD and TPF to the integrative analysis of OSCC gene expression and the microRNA-mRNA network. We identified five microRNAs that could be putative biomarkers for OSCC. Among them, one has been reported as biomarker and two are reported as associated microRNAs. The other two are the novel finding microRNA biomarkers. As a result, four novel biomarker microRNAs, that is, miR-148b-3p, miR-629-3p, miR-27a-3p, and miR-142-3p, are discovered in our work. The literature checking and the functional enrichment analysis confirmed our finding. Therefore, further experimental verification and clinical testing were suggested for these putative OSCC microRNA biomarkers.

## Figures and Tables

**Table 1:** OSCC gene expression data collected from GEO database.

Accession/ID	PMID	Platform	OSCC	Control	Gene/microRNA
GSE3524	15381369	GPL96	*n* = 16	*n* = 4	Gene
GSE70604	26700817	GPL2986	*n* = 7	*n* = 6	Gene
	23624915				
GSE37991	25204733	GPL6883	*n* = 40	*n* = 40	Gene
	23362108				
**GSE28100**	**22761427**	**GPL10850**	**n** = **17**	**n** =** 3**	**MicroRNA**
GSE23558	22072328	GPL6480	*n* = 27	*n* = 5	Gene
GSE25099	21853135	GPL5175	*n* = 57	*n* = 22	Gene
**GSE30784**	**18669583**	**GPL570**	**n** =** 167**	**n** =** 45**	**Gene**
GSE10121	18472963	GPL6353	*n* = 35	*n* = 6	Gene

**Table 2:** Previously reported microRNA biomarkers for OSCC from PubMed.

MicroRNA	PMID	Biomarker Type	Samples	Expression in OSCC	NOD (Ref.)^a^	TFP (Ref.)^a^
miR-155-5p	26307116 24692283	Prognostic biomarker	Tissue	Upregulated	71	0.211

miR-483-5p	26224475	Diagnostic/prognostic biomarker	Serum	Upregulated	4	0.135

miR-216a	25955794	Prognostic biomarker	Tissue	Downregulated	6	0.087

miR-21-3p miR-96-5p miR-141-3p miR-130b-3p	25532855	Prognostic biomarker	Tissue	Upregulated	N/A 8 22 2	N/A 0.159 0.167 0.119

miR-21-5p	24755828	Prognostic biomarker	Tissue	Upregulated	38	0.135

miR-31-5p	22083872	Diagnostic biomarker	Saliva	Upregulated	10	0.142

^a^The NOD and TFP are calculated based on the reference human microRNA-mRNA network, while the measurements in Section 3.2 are calculated based on the OSCC-specific network.

**Table 3:** Candidate OSCC microRNA biomarkers identified by our model.

MicroRNA	NOD	*p* value	TFP	*p* value	Number of targets
miR-148b-3p	15	5.06E-06	0.168	0.00912	95
**miR-155-5p**	**27**	**1.25E − 12**	**0.259**	**1.53E − 05**	**58**
miR-629-3p	10	0.00845	0.214	4.58*E* − 05	14
miR-27a-3p	10	0.00845	0.194	3.81*E* − 04	31
miR-142-3p	10	0.00845	0.164	0.0168	55

## Abbreviations

OSCC: Oral squamous cell carcinoma
TF: Transcription factor
NOD: Novel out degree
TFP: Transcription factor gene percentage
KEGG: Kyoto Encyclopedia of Genes and Genomes
DAVID: Database for Annotation, Visualization, and Integrated Discovery
IPA: Ingenuity Pathway Analysis.
