# Corrigendum to “Biomarker MicroRNAs for Diagnosis of Oral Squamous Cell Carcinoma Identified Based on Gene Expression Data and MicroRNA-mRNA Network Analysis”

**DOI:** 10.1155/2017/1284606

**Published:** 2017-12-28

**Authors:** Hui Zhang, Tangxin Li, Linqing Zheng, Xiangya Huang

**Affiliations:** ^1^Guanghua School of Stomatology, Hospital of Stomatology, Sun Yat-sen University, Guangdong Province Key Laboratory of Stomatology, Guangdong, China; ^2^Oral and Maxillofacial Center, Kiang Wu Hospital, Macau

In the article titled “Biomarker MicroRNAs for Diagnosis of Oral Squamous Cell Carcinoma Identified Based on Gene Expression Data and MicroRNA-mRNA Network Analysis” [1], there was a mistake in the first affiliation. The corrected affiliations are shown above.
